# Rapid response of thyroid eye disease, peripheral edema, and acropathy to teprotumumab infusion

**DOI:** 10.1016/j.ajoc.2024.102031

**Published:** 2024-03-02

**Authors:** David A. Kelly, Roger E. Turbin

**Affiliations:** Rutgers New Jersey Medical School, Department of Ophthalmology & Visual Science, 90 Bergen St. Ste. 6100, Newark, NJ, 07103, USA

**Keywords:** Thyroid eye disease, Graves disease, Thyroid dermopathy, Peripheral edema, Acropathy, Teprotumumab

## Abstract

**Purpose:**

We present a case of rapid improvement in symptoms of thyroid eye disease and amelioration of worsening peripheral edema and acropathy with infusion of teprotumumab, a monoclonal antibody targeting the insulin-like growth factor-1 receptor.

**Observations:**

A 66 year old female with history of Hashimoto thyroiditis developed progressive thyroid eye disease (TED), peripheral edema, and acropathy attributable to acute Graves disease. Her signs and symptoms, refractory to oral steroid and diuretic therapy, rapidly improved following a standard dosing regimen of teprotumumab (one infusion 10 mg/kg then seven infusions 20 mg/kg) to resolution.

**Conclusions & importance:**

Teprotumumab, a monoclonal antibody targeting the insulin-like growth factor-1 receptor, is the first medication approved by the FDA for use in TED. Teprotumumab may contribute to the treatment of extraocular manifestations of Graves disease, chief among these peripheral soft tissue manifestations.

## 1 Introduction

Graves disease is an uncommon autoimmune condition in which circulating thyroid stimulating autoantibodies interact with the thyroid stimulating hormone receptor, characteristically precipitating hyperthyroidism. Extrathyroidal manifestations result when these autoantibody-receptor complexes activate biochemical pathways in other organ systems, most notably activation of orbital fibroblasts in thyroid eye disease (TED). Similarly, peripheral soft tissue changes may occur in association with Graves disease. These include thyroid dermopathy, also known as pretibial myxedema, and acropachy, characterized by bony changes precipitated by chronic soft tissue inflammation at the joints of the hands and feet.^1^^,^^2^ There are significant limitations associated with established nonsurgical therapies for these extrathyroidal manifestations.^3^ Teprotumumab, a monoclonal antibody targeting the insulin-like growth factor-1 receptor (IGF-1R), competitively inhibits binding to this receptor on target tissues, thus decreasing downstream biochemical signaling and reducing the effect of circulating autoantibodies on these tissues.^4^ Teprotumumab is the first medication FDA approved for use in TED. Case reports have demonstrated response of thyroid dermopathy to the standardized dosing regimen.^5^, ^6^, ^7^, ^8^ We present a case of rapid improvement in symptoms of TED and amelioration of worsening peripheral edema and acropathy (not to be confused with acropachy) after treatment with teprotumumab.

## 2 Case report

**Fig. 1:**
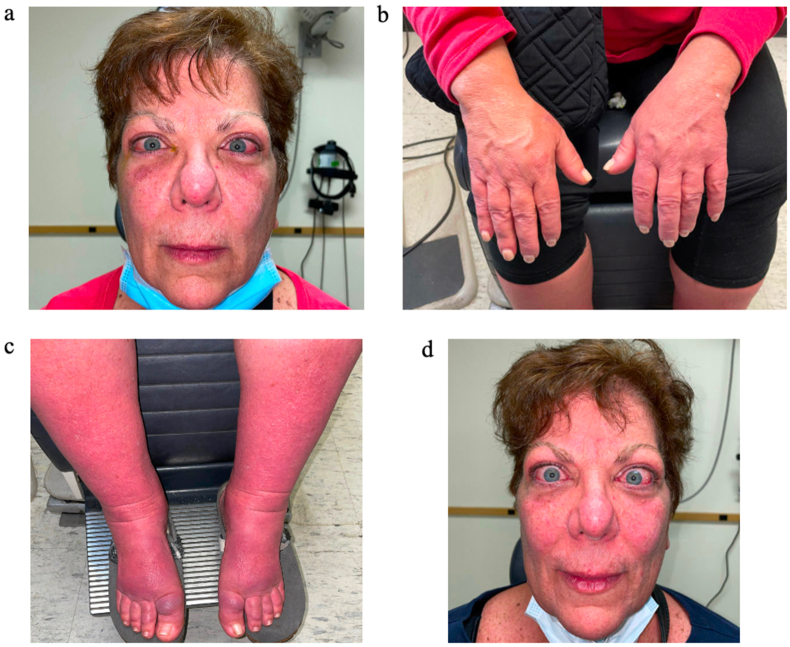
(a–c) This patient's acute thyroid eye disease was progressive six weeks prior to presentation in our office (then on day seven of prednisone therapy). She had significant orbital congestive symptoms including diplopia in addition to evident exacerbation of preexisting peripheral edema and acropathy. (d) On day 28 of prednisone therapy without notable response.

A 66 year old nonsmoking female with a previous history of Hashimoto thyroiditis developed acute Graves disease. She was previously maintained by her endocrinologist on liothyronine 25 mcg and furosemide 20mg daily for hypothyroidism and associated peripheral edema, respectively. She developed marked orbital symptoms, peripheral edema, and acropathy after influenza infection approximately five weeks prior to oculoplastic consultation. She presented to the service describing recent development of exophthalmos, progressive bilateral upper and lower eyelid edema, eyelid retraction, conjunctival erythema, chemosis, eye pain at rest and with extraocular movements, and intermittent diplopia accompanied by dramatic worsening of lower > upper extremity edema. Her lower extremity edema was so severe that she could only fit into specialized medical footwear. On exam she had marked orbital congestive signs and symptoms as noted above, severe generalized upper and lower extremity edema, and edema in the digits of her hands and feet (Fig. 1a–c). Hertel exophthalmometry measurements were 27mm on the right and 25mm on the left, base 110mm. Recent endocrine assessment included a thyroid stimulating hormone (TSH) 0.19 μU/mL (0.35–4.94 μU/mL), thyroid stimulating immunoglobulin (TSI) 7.73 IU/L (0.00–0.55 IU/L), thyrotropin receptor antibody (TRAb) > 40.00 IU/L (≤2.00 IU/L), and thyroid peroxidase antibody (anti-TPO) >1219 U/mL (0–60 U/mL).

**Fig. 2:**
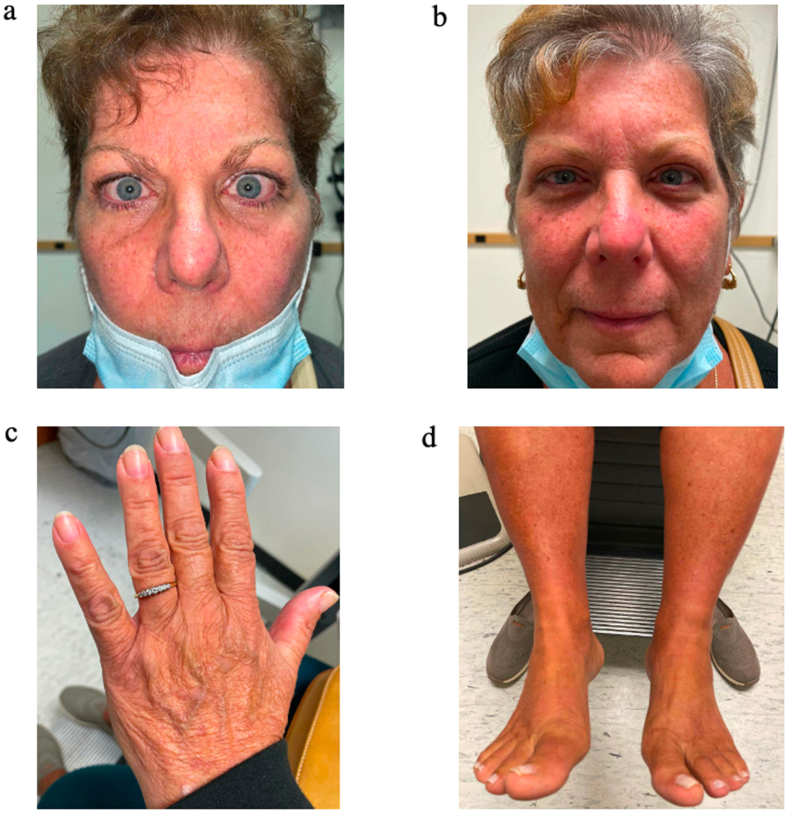
(a) Improvement in orbital symptoms after dose two of teprotumumab. Of note, prednisone was tapered off beginning day prior to first infusion and was discontinued 13 days later. (b–d) Dramatic improvement of thyroid eye disease, peripheral edema, and acropathy after full regimen of eight teprotumumab infusions. The patient reported complete resolution of symptoms including diplopia.

The management of this acute episode initially included cessation of liothyronine, institution of oral prednisone 10mg daily (increased to 40mg daily six days later), and escalation of oral furosemide dosing to 80mg BID without significant benefit. She was examined again following 28 days of treatment with this regimen without significant change in physical findings and symptoms (Fig. 1d). The following day she received the first infusion of teprotumumab and simultaneously began a taper of prednisone, which was discontinued entirely within 13 days. Subsequent physical examination 34 days into the teprotumumab course (after the second infusion) documented dramatically improved orbital and peripheral signs and symptoms (Fig. 2a). Thyroid supplementation for her baseline hypothyroidism was resumed around this time and resolution of all ocular and peripheral symptoms including diplopia was documented by her fifth infusion and maintained through her final infusion (Fig. 2b–d). Repeat Hertel exophthalmometry measurements after the final infusion were 17mm on the right and 16mm on the left, base 97mm.

She initially received sporadic infusions of IV furosemide approximately once weekly for control of midbody (not peripheral) edema until she completed a full regimen of eight infusions of teprotumumab. She reported that adverse effects of the teprotumumab included mild to moderate headache, lower extremity cramping, and dry eyes, each easily controlled with conservative management. She remains euthyroid on supplementation and no longer requires furosemide over a year after her final infusion.

## 3 Discussion

In this single case, teprotumumab infusion led to rapid improvement and ultimate resolution of medically refractory TED, peripheral edema, and acropathy associated with Graves disease. In the phase three study of the medication's indication for thyroid eye disease, 56% of patients in the treatment group achieved orbital response (as defined by at least 2mm reduction in proptosis in one eye) at six weeks.^9^ Our patient had dramatic improvement in both orbital signs and symptoms of TED as well as the exacerbation of peripheral soft tissue changes by two weeks after the second infusion, 34 days into IGF-1 receptor blockade institution. Improvements appear durable 13 months after treatment completion.

Graves disease is thought to effect TED by stimulation of orbital fibroblasts, which promotes glycosaminoglycan deposition within orbital structures. A similar mechanism may effect thyroid dermopathy; several previous reports demonstrate response of dermopathy to IGF-1R inhibition.^5^, ^6^, ^7^, ^8^ Our patient had preexisting peripheral edema diagnosed as a component of Hashimoto thyroiditis which severely worsened during the Graves episode. While the characterization of this worsening edema is incomplete in the absence of formal dermatologic and radiographic evaluation, photographic documentation is presented. The patient's nonpitting peripheral edema and the characteristic peau d’ orange texture of the skin suggest thyroid dermopathy. Her visible clubbing of the fingers also suggests digital soft tissue inflammation. Without assessment for characteristic radiographic evidence, bony changes indicating chronic acropachy cannot be confirmed.

TED frequently presents with for 12–24 month inflammatory phase prior to a period of quiescence; this pattern is independent of a patient's treatment toward euthyroid status.^4^ The rapid reversal of worsening followed by sustained improvement in previously refractory edema supports teprotumumab's efficacy in extra-orbital manifestations of Graves disease. Prior to administration of teprotumumab this patient's edema continued to worsen despite steroid and diuretic therapy. Amelioration of both TED and extra-orbital manifestations of Graves disease began immediately with teprotumumb infusion and the response appears durable. There are no FDA approved disease modifying therapies for extraorbital manifestations of Graves disease, but this case report adds to a growing body of evidence supporting the role of teprotumumab in treating these manifestations.

## 4 Patient consent

The patient consented in writing to the publication of this case and consented in writing to the use of clinical photographs in publication, including necessarily identifying photographs.

## CRediT authorship contribution statement

**David A. Kelly:** Writing – review & editing, Writing – original draft, Investigation. **Roger E. Turbin:** Writing – review & editing, Writing – original draft, Supervision, Project administration, Investigation, Conceptualization.

## Declaration of competing interest

No conflict of interest exists.
